# Direct Patterning of Carbon Nanotube via Stamp Contact Printing Process for Stretchable and Sensitive Sensing Devices

**DOI:** 10.1007/s40820-019-0323-8

**Published:** 2019-10-23

**Authors:** Binghao Liang, Zian Zhang, Wenjun Chen, Dongwei Lu, Leilei Yang, Rongliang Yang, Hai Zhu, Zikang Tang, Xuchun Gui

**Affiliations:** 1 State Key Laboratory of Optoelectronic Materials and Technologies, School of Electronics and Information Technology, Sun Yat-sen University, Guangzhou, 510275 People’s Republic of China; 2 State Key Laboratory of Optoelectronic Materials and Technologies, School of Physics, Sun Yat-Sen University, Guangzhou, 510275 People’s Republic of China; 3 Institute of Applied Physics and Materials Engineering, University of Macau, Avenida da Universidade, Taipa, Macau, People’s Republic of China

**Keywords:** Carbon nanotube, Strain sensor, Dry transfer, Stamp contact printing process

## Abstract

Flexible and wearable sensing devices have broad application prospects in bio-monitoring such as pulse measurement, motion detection and voice recognition. In recent years, many significant improvements had been made to enhance the sensor’s performance including sensitivity, flexibility and repeatability. However, it is still extremely complicated and difficult to prepare a patterned sensor directly on a flexible substrate. Herein, inspired by typography, a low-cost, environmentally friendly stamping method for the mass production of transparent conductive carbon nanotube (CNT) film is proposed. In this dry transfer strategy, a porous CNT block was used as both the seal and the ink; and Ecoflex film was served as an object substrate. Well-designed CNT patterns can be easily fabricated on the polymer substrate by engraving the target pattern on the CNT seal before the stamping process. Moreover, the CNT film can be directly used to fabricate ultrathin (300 μm) strain sensor. This strain sensor possesses high sensitivity with a gauge factor (GF) up to 9960 at 85% strain, high stretchability (> 200%) and repeatability (> 5000 cycles). It has been used to measure pulse signals and detect joint motion, suggesting promising application prospects in flexible and wearable electronic devices.

**Electronic supplementary material:**

The online version of this article (10.1007/s40820-019-0323-8) contains supplementary material, which is available to authorized users.

## Highlights

- A dry transfer method for the mass production of transparent conductive carbon nanotube (CNT) films inspired by typography has been proposed.
- The strain sensors based on the CNT films have high stretchability and repeatability (gauge factor up to 9960 at 85% strain).
- These ultrathin strain sensors can detect human motion, sound, and pulse, suggesting promising application prospects in wearable devices.

**Electronic supplementary material:**

The online version of this article (10.1007/s40820-019-0323-8) contains supplementary material, which is available to authorized users.

## Graphical abstract

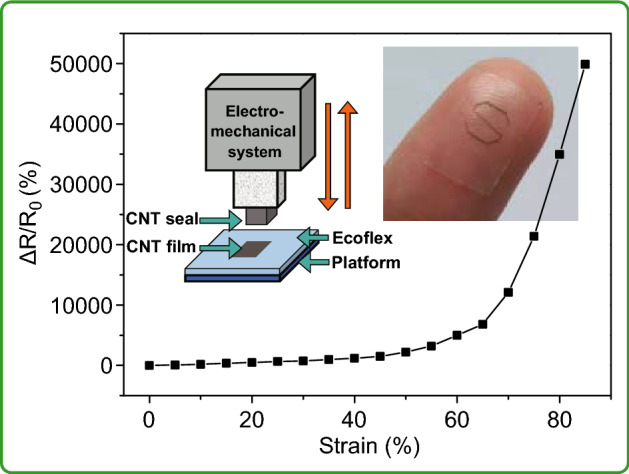

## Introduction

Flexible electronic devices have a wide range of needs and attracted tremendous attention in wearable electronics, soft robots and implantable medical devices [1–5]. Among the various functions of flexible electronic devices, strain sensing is the most fundamental and indispensable one [6–9]. These applications require the strain sensor to be ultrathin, transparent, integrative and easy to fabricate. Besides, the strain sensor also needs to be flexible and conformable for electronic skins or wearable electronics [10–12]. Generally, the performance of the strain sensor including sensitivity, stability, and respond speed depends not only on the stretchable substrate but also on the conductive network which transforms the strain from deformation into an electrical signal [13–15]. Despite some sensors with remarkable performance have been fabricated by fancy structural design [16, 17], or special materials modified [18], high-efficiency, low-cost and environmentally friendly manufacturing strategy for embedding conductive materials into polymer substrate are still one of the technological difficulties in fabricating strain sensors [19–23].

Recently, some methods have been developed for constructing a conductive network on stretchable polymer substrate. For instance, conductive nanomaterials were first dispersed in organic solutions and deposited on a polymer substrate by spin-coating [24–26], dip-coating [27, 28], or printing technology [21, 29–31]. Although many sensors fabricated by these technologies possess high sensitivity and larger stretchability, the dispersion process of conductive nanomaterials will destroy their structure and lead to an enormous decrease in conductivity. In addition, most of the organic solutions can damage the structure of the polymer substrate, which will lower the stretchability of the devices. Therefore, dry transfer, which can reduce the structural failure of conductive materials, is a feasible strategy for strain sensor fabrication. Recently, some new dry transfer methods have been explored for the fabrication of wearable devices. For example, Qiao et al. [32] demonstrated graphene epidermal artwork sensors based on laser scribed graphene. Gilshteyn et al. [33] developed a one-step technique to transfer CNT films on to hydrogel surface. Liao et al. [34] fabricated graphite-based strain sensors by pencil drew on printing paper. However, the above fabricating technologies are low-efficiency, high-cost, and complicated. A high-efficiency, eco-friendly, and low-cost method for fabricating strain sensors with excellent performance has not been explored, especially for the sensors with high sensitivity and stretchability.

Here, we proposed a simple, low-cost, environmentally friendly stamp contact printing method for the mass production of transparent conductive carbon nanotube (CNT) film. Stamp, similar to typography technology, has been widely used in official documents for nearly 3000 years. During the stamping process, the seal with well-designed pattern adsorbs and transfers liquid ink onto the target substrate. Afterward, the pattern will be printed on the substrate. Herein, inspired by stamp, a versatile stamp contact printing technology to prepare transparent CNT film on polymer substrate was developed. In this stamping method, a porous CNT block was used as both the seal and the solid ink. After the stamping process, the surface layer of CNT will be separated from the seal and transferred onto the Ecoflex surface with the help of the van der Waals’ interaction. The patterns on the CNT seal engraved by the laser can be transferred onto the target substrate and form a patterned CNT film. Further, we can fabricate strain sensors merely by connecting electrodes on both ends of the as-prepared CNT film. The strain sensor based on this CNT film shows not only high sensitivity and stretchability (gauge factor (GF) of 9959.8, at strain 85%), but also high repeatability (> 5000 cycles). Besides, even after stretching, bending and twisting for 1000 cycles, the resistance of the strain sensors had a tiny change, which shows the excellent recoverability of the CNT percolation network. To excavate the potential application of our sensors, pulse detection, motion monitoring, and voice recognition are demonstrated.

## Experimental

### Fabrication of CNT Film and Strain Sensor

The CNT seals were synthesized by chemical vapor deposition (CVD) method using ferrocene and 1,2-dichlorobenzene as the catalyst precursor and carbon source, as reported in our previous work [35]. The Ecoflex substrates were fabricated by mixing the A and B components of Ecoflex 00-30 (Smooth-On) rubbers in a volume ratio of 1:1 and coating on a glass substrate. After curing at room temperature for 10 h, the Ecoflex thin film, with a thickness of 300 μm, can be separated from the glass substrate. The CNT seal was fixed on the linear motor, which can move along z-axis and stamp the CNT seal on the Ecoflex substrate to form the CNT film. After the dry transfer process, UV/O_3_ treatment was used to enhance the interaction between CNT and the substrate. To fabricate the CNT strain sensor, two silver wires were connected on both ends of the CNT conductive film by silver conductive gel.

### Device Characterization

The morphology of the CNT block and transferred CNT film were characterized by Hitachi S-4800 field emission scanning electron microscope. A homemade system was used to measure the electromechanical performance of the strain sensor. This system consists of a digital multimeter (Keithley 2400) and a commercial linear mechanical motor (Zolix, TSA 300). Optical transmittances of CNT film were characterized by Ocean Optic Spectrometers (Maya 2000 Pro) together with a balanced deuterium halogen light source (Ocean Optics DH-2000-BAL).

## Result and Discussion

**Fig. 1:**
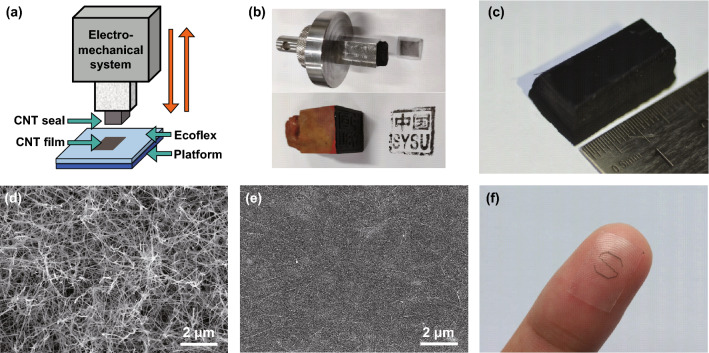
Schematic of the fabricating process and as-prepared samples of CNT films by stamp method. **a** Schematic illustration of the fabricating process. **b** Photograph of the CNT seal and stone seal. **c** Photograph of the CNT block. **d** SEM image of CNT block. **e** SEM image of the CNT film on Ecoflex substrate. **f** Transferred CNT film with an “S” pattern

The fabricating process of the CNT film is similar to typography, as schematically illustrated in Fig. 1a. A porous CNT block served as both the seal and solid ink, while an Ecoflex thin film was served as the target substrate. The CNT seal, which was mounted on the forcemeter, was pressed on the Ecoflex substrate and then released. A layer of the CNT in the CNT seal could be transferred onto the Ecoflex and formed a conductive CNT network on the surface of the polymer substrate. In this process, if the van der Waals’ interaction between this layer of CNT and the substrate is greater than the bonding strength between the CNT and the CNT seal, the layer of CNT can be transferred. After the dry transfer process, we can use ultraviolet (UV)/O_3_ treatment to further enhance the interaction and bonding strength between the CNT film and the polymer substrate. Through UV/O_3_ treatment, the surface of the silicone rubber could develop many polar groups [36, 37], which could improve wettability and adhesion of the substrate. The prepared CNT film is very uniform throughout the stamp range, similar to the stamp by a stone seal with the words of “SYSU” (Fig. 1b). The photograph of the CNT seal is shown in Fig. 1c. This CNT film preparing method is simple, high-efficiency, energy-saving and eco-friendly, which makes it suitable for mass production.

As discussed above, a successful stamp depends on both the seal and target substrate. The microstructure of the CNT seal has been characterized by scanning electron microscopy (SEM), as shown in Fig. 1d. It indicates that the CNT seal consists of CNT which were self-assembled into the anisotropic, porous and interconnected framework. This characteristic ensures that the transferred CNT film on the Ecoflex substrate has a homogeneous network structure. As shown in Fig. 1e, the transferred CNT film formed a uniformity network structure and embedded on the surface of the Ecoflex. Like the stamp, we can also engrave different words or patterns on the CNT seal by laser ablation to fabricate CNT films with any expected patterns. As a demonstration, a CNT word “S” was directly stamped on the Ecoflex substrate by an engraved CNT seal (Fig. 1f). The strong attachment of the sample to fingerprint shows the excellent conformability of our fabricated film, which is very favorable for the strain sensor.

**Fig. 2:**
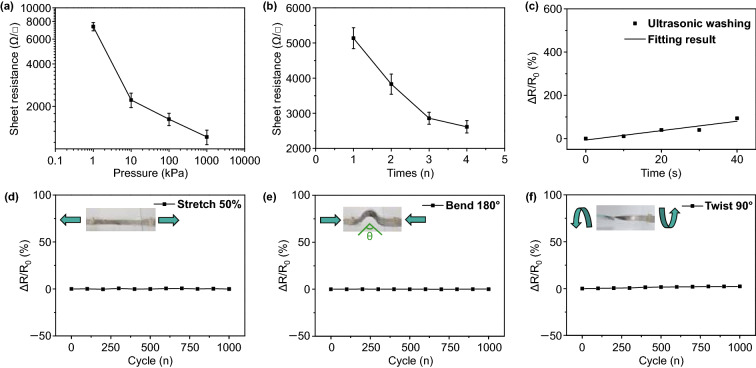
Characterization of the electrical properties of CNT films. The sheet resistance of the transferred CNT films **a** at different stamp pressure, **b** at different stamp number of times. **c** The change of sheet resistance of the CNT film after ultrasonic washing at different time. Electric stability of CNT film **d** during 1000 cycles stretching under strain of 50%, **e** during 1000 cycles under bending (180 degrees), **f** during 1000 cycles twisting (90 degrees)

The performance of the transferred CNT film depends on the technological parameters in the transfer process and the mechanical property of the CNT block. By adjusting the stamp pressure, we can control the thickness of the transferred CNT film. With the increase in stamp pressure, the thickness of the CNT film will be increased, resulting in a decrease in sheet resistance (Fig. 2a) and transmittance (Fig. S1). For the sample fabricated at the stamp pressure of 100 kPa, the sheet resistance and transmittance at 550 nm were about 1.8 kΩ/□ and 50%, respectively. Because Young’s modulus of the porous CNT seal (10 mg cm^−3^) is relatively low [35], the CNT could be contacted with the Ecoflex substrate more easily. The bonding strength between the CNT and the substrate will become larger with the increase in stamp pressure; therefore, more CNT will be transferred onto the substrate. As a comparison, a CNT block with higher Young’s modulus (density of 100 mg cm^−3^) was used as a seal. The CNT film fabricated by this seal has higher sheet resistance 4 kΩ/□ at the same stamp pressure (100 kPa) (Fig. S2). The CNT seal also acts as a solid ink. In each stamp, the thickness of the CNT film is only less than two hundred nanometers [38]. The performance and microstructure of the transferred CNT film have no significant change during repeated stamp. The sheet resistance increased only about 2 times after 30 times stamping (Fig. S3). If the stamp is repeated on the same position in the substrate, the resistance of the obtained CNT film will decrease and finally stabilize. As shown in Fig. 2b, the sheet resistance drops dramatically from the initial 5–2.5 kΩ/□. Compared with some recent dry transfer methods [33, 39, 40], our typography-inspired dry transfer method has advantages in controlling the thickness of the conductive layer, lower the cost and reduce the pollution in mass production.

The transferred CNT films have strong interaction with the substrate. Even under ultrasonic washing, most of the CNT still stick on the surface of the Ecoflex substrate. The resistance of the sample only increases by less than 50% after 40 s of ultrasonic washing, as shown in Fig. 2c. This can be further confirmed during the second transfer. By using a fresh Ecoflex to dry transfer the as-prepared CNT/Ecoflex thin film, there was a tiny change in the resistance of the as-prepared CNT film (Fig. S4b). Van der Waals’ force and hydrogen bonding forces are the main interaction between the transferred CNT and substrate, which can be enhanced by UV/O_3_ treatment. As the UV/O_3_ treatment time increases, the resistance of the CNT film decreases, indicating that the connection between the CNT is better (Fig. S4a). UV/O_3_ treatment strengthens both the CNT–CNT interaction and CNT-Ecoflex interaction. Because of the strong interaction between CNT film and Ecoflex substrate, the electrical properties of the CNT films are stable. After the CNT film was stretched (*ε* = 50%), bent (*θ* = 180°) and twisted (*θ* = 90°) for 1000 cycles, its sheet resistance has tiny change (Fig. 2d–f). This indicates that the CNT conductive network is stable with little displacement and fracture.

**Fig. 3:**
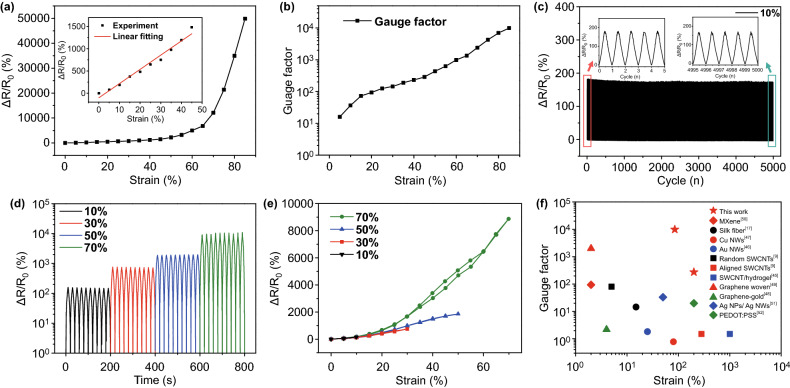
Sensing properties of CNT-based strain sensors. **a** Relative resistance response of CNT-based strain sensors versus applied strain. **b** Gauge factor of CNT-based strain sensors versus applied strain. **c** Relative resistance change versus applied strain over 5000 cycles. **d** Relative resistance change versus applied strain with increasing maximum strain from 10% to 70%. **e** Hysteresis curves of the stretch/release cycles under different maximum applied strain. **f** Comparison on gauge factor (GF) and maximum strain between our strain sensors and the recently reported strain sensors, which constructed by different conductive materials, including MXene [50], Silk fiber [17], Cu Nanowires (NWs) mesh [47], Au NWs [46], Single-walled carbon nanotubes (SWCNTs) [9], SWCNT/hydrogel [45], graphene woven microfabrics [49], graphene-gold mesh [48], Ag Nanoparticles (NPs)/Ag NWs [51] and PEDOT:PSS [52]

After silver electrodes were connected at both ends of the transferred CNT film, it can be directly used as a strain sensor. The relative resistance change *versus* applied strain of the sensor is shown in Fig. 3a. It shows two linear ranges in the resistance–strain curve, one is at the strain from 0 to 45%, and the other is from 45 to 85%. The sensing mechanism of the CNT-based strain sensor is illustrated in Fig. S5. The disconnection and reconnection of CNT junctions during the stretching/releasing cycles played a decisive role in the change of relative resistance. Under applied strain less than 45%, most of the CNT remain integrity, but some CNT could rotate to the axis of stretching. Therefore, the change of resistance is mainly caused by the deformation of the CNT percolation network. Some intersections between CNT will be broken, which also results in an increase in resistance (Fig. S5a). As shown in the inset of Fig. 3a, the relative resistance *versus* applied strain suggests extraordinary linearity. When the applied strain is larger than 45%, some of the CNT will be fractured and the resistance will increase dramatically as shown in Fig. 3a. As a comparison, an aligned CNT array was used as a seal and a layer of aligned CNT conductive network was transferred on the Ecoflex surface. A strain sensor was fabricated based on this CNT film. If the direction of applied strain were perpendicular to the CNT arrangement, individual CNT was hard to tear down. The deformation only separates the CNT. Under applied strain, the distance between the adjacent CNTs will increase, and some of the CNTs will rotate to the axis of stretching and form conductive CNT bridges. As reported in our early work [41], the CNTs are still connected to each other by the CNT bridges, even the strain arrived 270%. Therefore, the sensitivity of the strain sensor with aligned CNT is much smaller than the one with random CNT, as shown in Fig. S6. The similar sensing mechanism of nanowires- and nanotubes-based strain sensor has been reported in our previous work [41] and several papers [26, 42, 43] published by other groups.

Furthermore, the thickness of the transferred CNT plays a very important role in the sensitivity of the strain sensor. Thinner CNT film possesses better flexibility and smaller sheet resistance change, compared to the thicker one under the same applied strain. Due to the thickness of CNT can be adjusted by controlling the transfer pressure or transfer times (Fig. S7), the sensitivity of the strain sensor can be improved by increasing the transfer pressure or repeating the dry transfer process. For example, under the same applied strain (80%), the relative resistance changes of strain sensor with higher transfer pressure (1000 kPa) are about 530 times larger than the one with lower transfer pressure (1 kPa). Besides, by repeating the stamping transfer process for four times, the sensitivity of the strain sensor can improve about 3 times.

The CNT-based strain sensor also possesses high sensitivity and large stretchability. As shown in Fig. 3b, the GF of the strain sensors is 9960 at 85% applied strain. The GF increases with the applied strain. This is mainly due to the more junctions of the CNT connection will be broken with the increase in strain (Fig. S5), resulting in the dramatic increase in resistance. The maximum tensile strain exceeds 200% (Fig. S7b). Besides, the CNT-based strain sensor exhibited high stability and excellent recoverability in 5000 stretching/releasing cycles (as shown in Fig. 3c). The dynamic response of the strain sensor is shown in Figs. 3d and S8 under different maximum applied strain. The repeatability was excellent during 10 stretching/releasing cycles under both small and large strain. It demonstrates that the relative resistance monotonically increases with the increasing applied strain (Fig. S9). Figure 3e shows a small drift in the stretching/releasing cycles. In the first few cycles, some unrecoverable damages will be caused in the CNT film. This small drift only occurred when we first extend the maximum working strain, which will be eliminated after a few cycles. As shown in Fig. S10, during 10 stretching/releasing cycles at 70% strain, there was no drift between each cycle and the hysteresis between loading and unloading was negligibly small. Besides, the conductivity of CNT can be fully recovered after releasing from strain up to 70%. Figure 3f illustrates the GF *versus* the maximum working strain of strain sensors using different materials such as CNT [9, 44, 45], nanowires [46, 47], graphene [48, 49], and other conductive materials [27, 50–52]. Some of these strain sensors possess high sensitivity while others can withstand the large strain. However, few sensors can work under 50% strain with GF larger than 100. By controlling the thickness of CNT film, our strain sensors can work under 85% strain with GF up to 9960, and under 200% strain with GF up to 274.

**Fig. 4:**
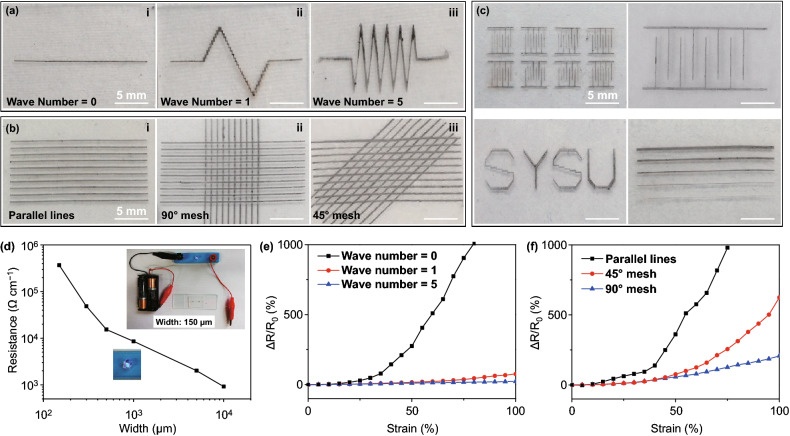
Transferred CNT films with different patterns and their electromechanical properties. **a** Optical camera images of wavy CNT strip with different wavenumbers. **b** Transferred CNT film with different mesh structures. **c** The different patterns of CNT network including an interdigital electrode, characters and strips with different line width. **d** The resistance of CNT strips with different line width. **e** Electromechanical properties of the strain sensor with different wave number pattern. **f** Electromechanical properties of the strain sensor with different direction mesh patterns

The patterns of CNT films also affect the sensitivity of the strain sensor. To attain strain sensors with different patterns or strain sensor matrix, CNT block was first patterned by laser scribe and this pattern was transferred directly onto the polymer substrate. In addition, we can also cover the polymer substrate by a patterned mask before the stamp. Therefore, well-designed CNT circuits can be easily fabricated on polymer substrate. With single time laser scribe, we can fabricate dozens of CNT-based strain sensors with particularly designed patterns. As shown in Fig. 4a-c, different patterns of CNT including wave, mesh, interdigital electrode, and character were printed on Ecoflex substrate. The limiting resolution of line width is about 150 μm, as shown in Fig. 4d, two batteries can light up the LED through the transferred CNT strips with 150 μm line width. The relationship between CNT strips width and the resistance is shown in Figs. 4d and S11. The sensing properties of the strain sensor also can be tuned by the pattern of the CNT film. To figure out how the CNT patterns affect the electronic properties of the strain sensors, serval different patterns of CNT film were fabricated as shown in Fig. 4a, b. Electrodes were connected at both ends of these patterns, and the tensile strain was applied in the horizontal direction. As shown in Fig. 4e, CNT-based strain sensor with different numbers of waves had different electromechanical performances. If there are no waves in the CNT network (Fig. 4a (i)), the resistance will change dramatically even under small applied strain. With the increase in wave numbers, the stretchability of the CNT film will increase (Fig. 4a (iii)). As shown in Fig. 4f, the sensitivity of the strain sensor with only parallel CNT pattern is much higher than the strain sensor with both parallel and vertical CNT networks, which shows that the vertical connection can enhance the flexibility of the strain sensor.

**Fig. 5:**
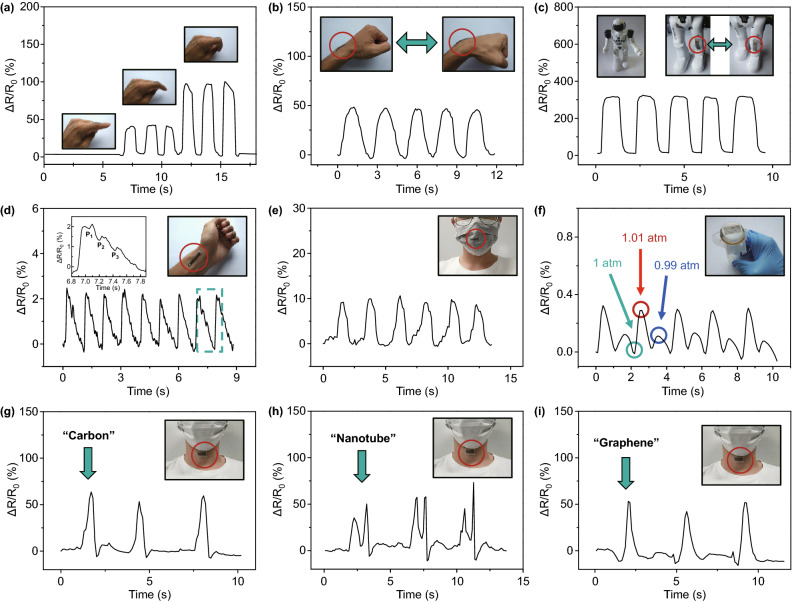
Several applications of CNT-based strain sensor. **a** Resistance response of finger motion. **b** Resistance response of wrist motion. **c** Monitoring the movement of a robot knee. **d** The pulse signal of a volunteer. **e** Detection of human breath through a mask. **f** Resistance response of tiny change of air pressure. **g** Monitoring the human voice with the word “Carbon”.**h** Monitoring the human voice with the word “Nanotube”. **i** Monitoring the human voice with the word “Graphene”

Several applications of the strain sensor are demonstrated in Fig. 5. The strain sensor can detect several kinds of human motion. Because of the small Young’s modulus and the ultrathin structure, the strain sensor has perfect adhesion and comfortability to human skin. In Fig. 5a, the strain sensor can be attached to a human finger, the resistance changes for different levels of finger bending. Figure 5b demonstrates joint motion monitoring in the human wrist. During the five bending and stretching cycles, the resistance response was stable and repeatable. Besides human motion detection, the strain sensor can also apply to robots as shown in Fig. 5c. A tiny signal such as pulse can be measured by our strain sensor as shown in Fig. 5d. It is clearly shown that three kinds of pulse wave including percussion wave, tidal wave, and diastolic wave can be well distinguished by our strain sensor. In addition, we can monitor the respiration signal by installing the strain sensor on a respirator as shown in Fig. 5e. When we seal a pasty test tube with our strain sensor, it can detect tiny change of air pressure. As shown in Fig. 5f, with 1% change of air pressure, the output signal of our strain sensor was distinguishable, and the response is quite stable during 5 cycles. In Fig. 5g–i, the strain sensor was attached on the throat of a volunteer; the received electronic signals were different due to the different pronunciation of words (“carbon,” “nanotube” and “graphene”). The as-mentioned application shows the great potential of our strain sensor in flexible devices for motion detection, health monitoring, and voice recognition.

## Conclusions

In this paper, we have developed a simple and environmentally friendly method via stamp contact printing process for the fabrication of stretchable and sensitive patternable carbon nanotube sensing devices. This dry transfer technique provides new insight and has a promising future in the mass production of the strain sensor. The strain sensor possesses impressively high sensitivity (GF up to 9960) in a wide range of applied strain. Besides, the strain sensors can detect tiny signals such as pulse, breath, and voice as well as larger deformation such as finger motion and robot movement. It suggested that this typography-inspired dry transfer method can be extended to other conductive materials and other substrates.

## Electronic supplementary material

Below is the link to the electronic supplementary material.

Supplementary material 1 (PDF 481 kb)

## References

[1] Chen Z, Wang Z, Li X, Lin Y, Luo N (2017). Flexible piezoelectric-induced pressure sensors for static measurements based on nanowires/graphene heterostructures. ACS Nano.

[2] Shi J, Wang L, Dai Z, Zhao L, Du M (2018). Multiscale hierarchical design of a flexible piezoresistive pressure sensor with high sensitivity and wide linearity range. Small.

[3] Wan C, Chen G, Fu Y, Wang M, Matsuhisa N, Pan S (2018). An artificial sensory neuron with tactile perceptual learning. Adv. Mater..

[4] Bai S, Zhang S, Zhou W, Ma D, Ma Y (2017). Laser-assisted reduction of highly conductive circuits based on copper nitrate for flexible printed sensors. Nano-Micro Lett..

[5] Boutry CM, Kaizawa Y, Schroeder BC, Chortos A, Legrand A (2018). A stretchable and biodegradable strain and pressure sensor for orthopaedic application. Nat. Electr..

[6] Amjadi M, Kyung KU, Park I, Sitti M (2016). Stretchable, skin-mountable, and wearable strain sensors and their potential applications: a review. Adv. Funct. Mater..

[7] Amjadi M, Pichitpajongkit A, Lee S, Ryu S, Park I (2014). Highly stretchable and sensitive strain sensor based on silver nanowire–elastomer nanocomposite. ACS Nano.

[8] Hempel M, Nezich D, Kong J, Hofmann M (2012). A novel class of strain gauges based on layered percolative films of 2D materials. Nano Lett..

[9] Yamada T, Hayamizu Y, Yamamoto Y, Yomogida Y, Izadi-Najafabadi A (2011). A stretchable carbon nanotube strain sensor for human-motion detection. Nat. Nanotechnol..

[10] Kong J, Jang N, Kim S, Kim J (2014). Simple and rapid micropatterning of conductive carbon composites and its application to elastic strain sensors. Carbon.

[11] Lu N, Lu C, Yang S, Rogers J (2012). Highly sensitive skin-mountable strain gauges based entirely on elastomers. Adv. Funct. Mater..

[12] Oh J, Yang J, Kim J, Park H, Kwon S (2018). Pressure insensitive strain sensor with facile solution-based process for tactile sensing applications. ACS Nano.

[13] Wu S, Peng S, Han Z, Zhu H, Wang C (2018). Ultrasensitive and stretchable strain sensors based on mazelike vertical graphene network. ACS Appl. Mater. Interfaces.

[14] Yang T, Jiang X, Zhong Y, Zhao X, Lin S (2017). A wearable and highly sensitive graphene strain sensor for precise home-based pulse wave monitoring. ACS Sens..

[15] Jiang Y, Liu Z, Matsuhisa N, Qi D, Leow WR (2018). Auxetic mechanical metamaterials to enhance sensitivity of stretchable strain sensors. Adv. Mater..

[16] Guo F, Cui X, Wang K, Wei J (2016). Stretchable and compressible strain sensors based on carbon nanotube meshes. Nanoscale.

[17] Zhang M, Wang C, Qi W, Jian M, Zhang Y (2016). Sheath-core graphite/silk fiber made by dry-meyer-rod-coating for wearable strain sensors. ACS Appl. Mater. Interfaces.

[18] Boland CS, Khan U, Ryan G, Barwich S, Charifou R (2016). Sensitive electromechanical sensors using viscoelastic graphene-polymer nanocomposites. Science.

[19] Son D, Kang J, Vardoulis O, Kim Y, Matsuhisa N, Oh J (2018). An integrated self-healable electronic skin system fabricated via dynamic reconstruction of a nanostructured conducting network. Nat. Nanotechnol..

[20] Wang C, Li X, Gao E, Jian M, Xia K (2016). Carbonized silk fabric for ultrastretchable, highly sensitive, and wearable strain sensors. Adv. Mater..

[21] Wang M, Wang W, Leow WR, Wan C, Chen G (2018). Enhancing the matrix addressing of flexible sensory arrays by a highly nonlinear threshold switch. Adv. Mater..

[22] Yan C, Wang J, Kang W, Cui M, Wang X (2014). Highly stretchable piezoresistive graphene–nanocellulose nanopaper for strain sensors. Adv. Mater..

[23] Sun X, Sun J, Li T, Zheng S, Wang C (2019). Flexible tactile electronic skin sensor with 3D force detection based on porous CNTs/PDMS nanocomposites. Nano-Micro Lett..

[24] Lee J, Kim S, Lee J, Yang D, Park BC (2014). A stretchable strain sensor based on a metal nanoparticle thin film for human motion detection. Nanoscale.

[25] Lu L, Wei X, Zhang Y, Zheng G, Dai K (2017). A flexible and self-formed sandwich structure strain sensor based on AgNW decorated electrospun fibrous mats with excellent sensing capability and good oxidation inhibition properties. J. Mater. Chem. C.

[26] Lipomi D, Vosgueritchian M, Tee B, Hellstrom S, Lee J (2011). Skin-like pressure and strain sensors based on transparent elastic films of carbon nanotubes. Nat. Nanotechnol..

[27] Cheng Y, Wang R, Sun J, Gao L (2015). A stretchable and highly sensitive graphene-based fiber for sensing tensile strain, bending, and torsion. Adv. Mater..

[28] Ge J, Yao H, Wang X, Ye Y, Wang J (2013). Stretchable conductors based on silver nanowires: improved performance through a binary network design. Angew. Chem. Int. Ed..

[29] Muth J, Vogt D, Truby R, Menga Y, Kolesky D (2014). Embedded 3D printing of strain sensors within highly stretchable elastomers. Adv. Mater..

[30] Michelis F, Bodelot L, Bonnassieux Y, Lebental B (2015). Highly reproducible, hysteresis-free, flexible strain sensors by inkjet printing of carbon nanotubes. Carbon.

[31] Zeng S, Zhang D, Huang W, Wang Z, Freire SG (2016). Bio-inspired sensitive and reversible mechanochromisms via strain-dependent cracks and folds. Nat. Commun..

[32] Qiao Y, Wang Y, Tian H, Li M, Jian J (2018). Multilayer graphene epidermal electronic skin. ACS Nano.

[33] Gilshteyn E, Lin S, Kondrashov V, Kopylova D, Tsapenko A (2018). A one-step method of hydrogel modification by single-walled carbon nanotubes for highly stretchable and transparent electronics. ACS Appl. Mater. Interfaces.

[34] Liao X, Liao Q, Yan X, Liang Q, Si H (2015). Flexible and highly sensitive strain sensors fabricated by pencil drawn for wearable monitor. Adv. Funct. Mater..

[35] Gui X, Wei J, Wang K, Cao A, Zhu H (2010). Carbon nanotube sponges. Adv. Mater..

[36] Ma K, Rivera J, Hirasaki GJ, Biswal SL (2011). Wettability control and patterning of PDMS using UV–ozone and water immersion. J. Colloid Interface Sci..

[37] Bhattacharya S, Datta A, Berg JM, Gangopadhyay S (2005). Studies on surface wettability of poly(dimethyl) siloxane (PDMS) and glass under oxygen-plasma treatment and correlation with bond strength. J. Microelectromech. Syst..

[38] Kaempgen M, Duesberg G, Roth S (2005). Transparent carbon nanotube coatings. Appl. Surf. Sci..

[39] Li Y, Zhang H, Yao Y, Li T, Zhang Y (2015). Transfer of vertically aligned carbon nanotube arrays onto flexible substrates for gecko-inspired dry adhesive application. RSC Adv..

[40] Pint C, Xu Y, Moghazy S, Cherukuri T, Alvarez N (2010). Dry contact transfer printing of aligned carbon nanotube patterns and characterization of their optical properties for diameter distribution and alignment. ACS Nano.

[41] Liang B, Lin Z, Chen W, He Z, Zhong J (2018). Ultra-stretchable and highly sensitive strain sensor based on gradient structure carbon nanotubes. Nanoscale.

[42] Zhou J, Yu H, Xu X, Han F, Lubineau G (2017). Ultrasensitive, stretchable strain sensors based on fragmented carbon nanotube papers. ACS Appl. Mater. Interfaces.

[43] Amjadi M, Yoon YJ, Park I (2015). Ultra-stretchable and skin-mountable strain sensors using carbon nanotubes-Ecoflex nanocomposites. Nanotechnology.

[44] De Vivo B, Lamberti P, Spinelli G, Tucci V (2014). Simulation and experimental characterization of polymer/carbon nanotubes composites for strain sensor applications. J. Appl. Phys..

[45] Cai G, Wang J, Qian K, Chen J, Li S (2016). Strain sensors: extremely stretchable strain sensors based on conductive self-healing dynamic cross-links hydrogels for human-motion detection. Adv. Sci..

[46] Gong S, Schwalb W, Wang Y, Chen Y, Tang Y (2014). A wearable and highly sensitive pressure sensor with ultrathin gold nanowires. Nat. Commun..

[47] Han S, Kim MK, Wang B, Wie DS, Wang S (2016). Mechanically reinforced skin-electronics with networked nanocomposite elastomer. Adv. Mater..

[48] Lee H, Choi TK, Lee YB, Cho HR, Ghaffari R (2016). A graphene-based electrochemical device with thermoresponsive microneedles for diabetes monitoring and therapy. Nat. Nanotechnol..

[49] Yang T, Wang W, Zhang H, Li X, Shi J (2015). Tactile sensing system based on arrays of graphene woven microfabrics: electromechanical behavior and electronic skin application. ACS Nano.

[50] Ma Y, Liu N, Li L, Hu X, Zou Z (2017). A highly flexible and sensitive piezoresistive sensor based on MXene with greatly changed interlayer distances. Nat. Commun..

[51] Kim HJ, Sim K, Thukral A, Yu C (2017). Rubbery electronics and sensors from intrinsically stretchable elastomeric composites of semiconductors and conductors. Sci. Adv..

[52] Lipomi DJ, Lee JA, Vosgueritchian M, Tee CK, Bolander JA, Bao Z (2012). Electronic properties of transparent conductive films of PEDOT: PSS on stretchable substrates. Chem. Mater..

