# Supplementary material for: Direct Patterning of Carbon Nanotube via Stamp Contact Printing Process for Stretchable and Sensitive Sensing Devices
Source: Nanomicro Lett. 2019 Oct 23;11:92. doi: 10.1007/s40820-019-0323-8 (PMC7770666; doi:10.1007/s40820-019-0323-8)
Supplement: Supplementary file 1 — Supplementary material 1 (PDF 481 kb) [file 40820_2019_323_MOESM1_ESM.pdf]

Supporting Information for

Direct Patterning of Carbon Nanotube *via* Stamp Contact Printing Process for Stretchable and Sensitive Sensing devices

Binghao Liang[1], Zian Zhang[1], Wenjun Chen[1], Dongwei Lu[1], Leilei Yang[1], Rongliang Yang[1], Hai Zhu[2], Zikang Tang[3], Xuchun Gui[1, *]

[1]State Key Laboratory of Optoelectronic Materials and Technologies, School of Electronics and Information Technology, Sun Yat-sen University, Guangzhou 510275, People's Republic of China

[2]State Key Laboratory of Optoelectronic Materials and Technologies, School of Physics, Sun Yat-Sen University, Guangzhou 510275, People's Republic of China

[2]Institute of Applied Physics and Materials Engineering, University of Macau, Avenida da Universidade, Taipa, Macau, People's Republic of China

*Corresponding author. E-mail: guixch@mail.sysu.edu.cn (Xuchun Gui)

Supplementary Figures

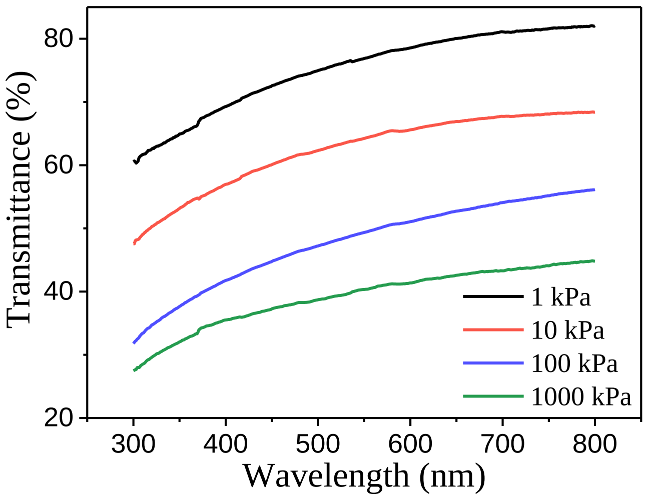

Fig. S1 Transmittance of the CNT films under different stamp pressure

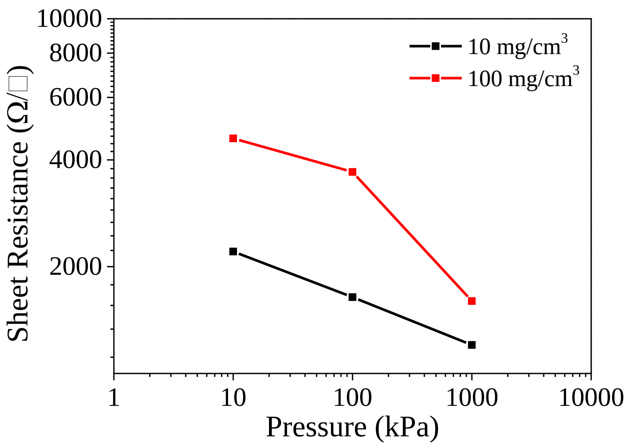

Fig. S2 Sheet resistance of CNT films fabricated using CNT seal with different density

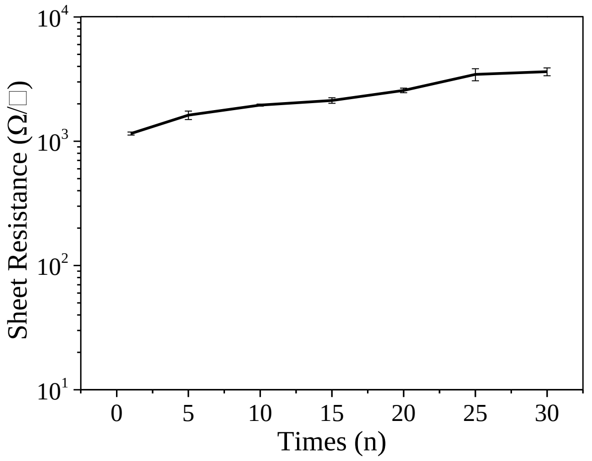

Fig. S3 Sheet resistance of different CNT films fabricated using the same CNT seal

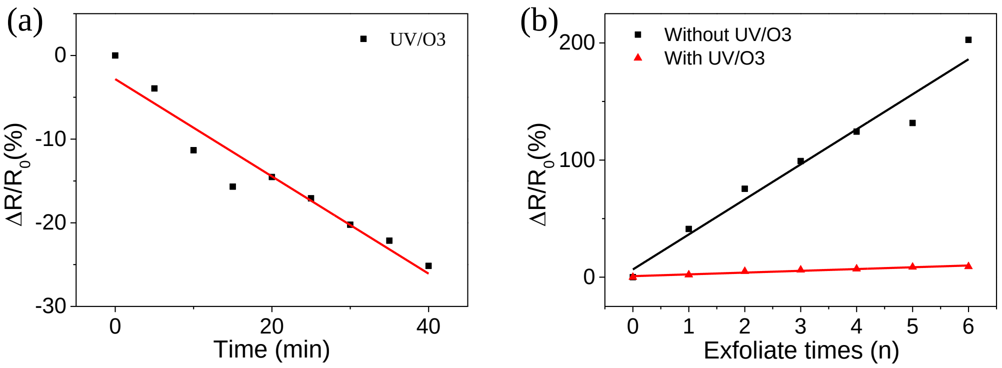

Fig. S4 Influence of UV/O_3 treatment on conductivity of CNT film. (**a**) Relative resistance changes of the same sample after different times of UV/O_3 treatment. (**b**) Relative resistance changes of the original CNT film after multiple transfers, in which a fresh Ecoflex covered on the original as-prepared CNT/Ecoflex thin film and peeled off to transfer CNT

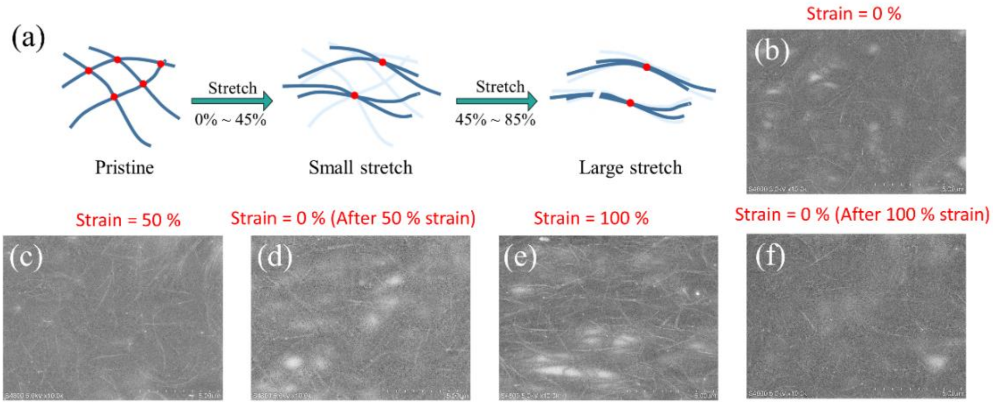

Fig. S5 Schematic and SEM images illustrated the sensing mechanism of the strain sensors. **(a)** Schematic illustration of the sensing mechanism of CNT-based strain sensor. **(b)** SEM image of CNT-based strain sensor before stretch. **(c)** SEM image of CNT-based sensor under stretching strain of 50%. **(d)** SEM image of CNT-based strain sensor released from 50% stretching strain. **(e)** SEM image of CNT-based strain sensor under stretching strain of 50%. **(f)** SEM image of CNT-based strain sensor released from 100% stretching strain

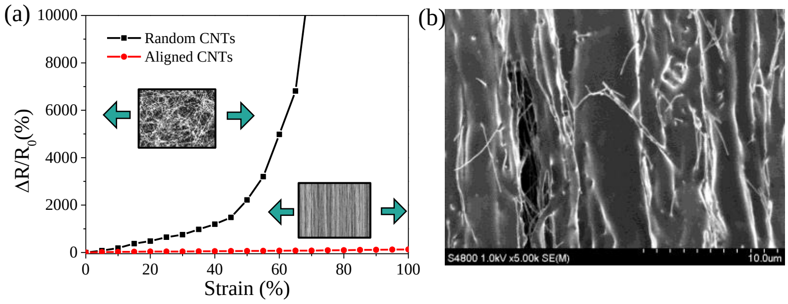

Fig. S6 (a) Comparison of CNT-based strain sensors fabricated by using random CNT seal and aligned CNT seal. **(b)** SEM image of the CNT array on Ecoflex substrate

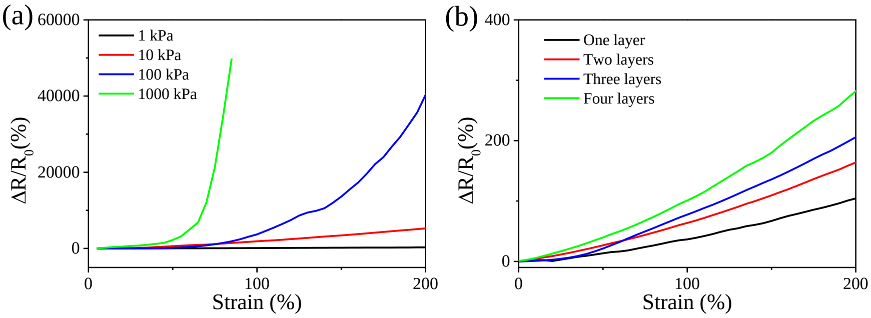

Fig. S7 Electromechanical properties of CNT-based strain sensors. **(a)** Comparison of CNT strain sensor fabricated by different stamp pressure. **(b)** Comparison of CNT strain sensor fabricated by different transfer times

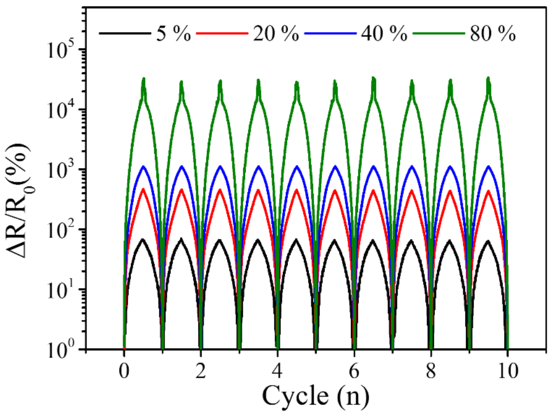

Fig. S8 The difference of relative resistance under different maximum applied strain

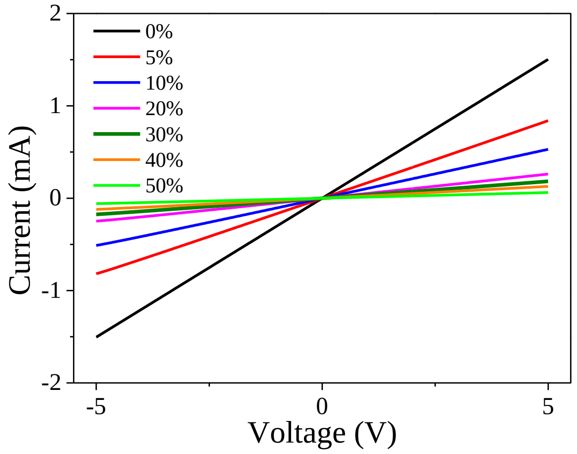

Fig. S9 *I/V* curves of CNT-based strain sensor under different stretching strain

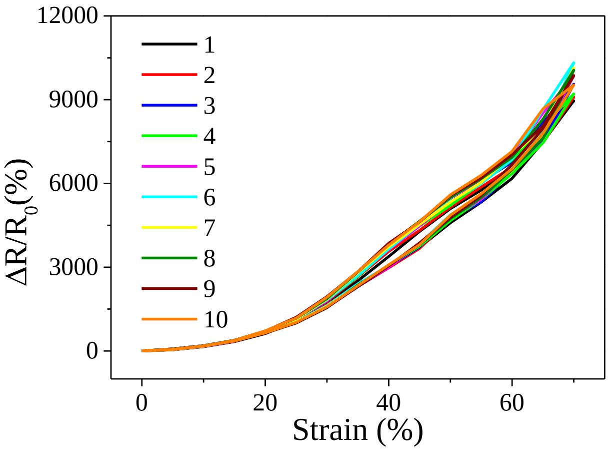

Fig. S10 Repeatability of CNT-based strain sensor for 10 stretching/releasing cycles under 70% applied strain

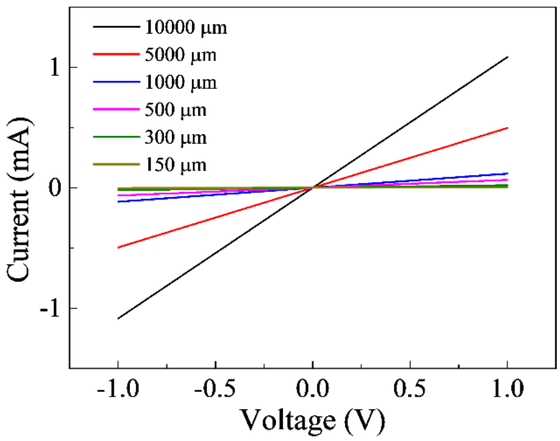

Fig. S11 *I*/*V* curves of CNT-based strain sensors with different CNT strips width
